# A reliability and validity study of the Palliative Performance Scale

**DOI:** 10.1186/1472-684X-7-10

**Published:** 2008-08-04

**Authors:** Francis Ho, Francis Lau, Michael G Downing, Mary Lesperance

**Affiliations:** 1 School of Health Information Science, University of Victoria, Victoria, British Columbia, Canada; 2 Victoria Hospice Society, Victoria, British Columbia, Canada; 3 Department of Mathematics and Statistics, University of Victoria, British Columbia, Canada

## Abstract

**Background:**

The Palliative Performance Scale (PPS) was first introduced in1996 as a new tool for measurement of performance status in palliative care. PPS has been used in many countries and has been translated into other languages.

**Methods:**

This study evaluated the reliability and validity of PPS. A web-based, case scenarios study with a test-retest format was used to determine reliability. Fifty-three participants were recruited and randomly divided into two groups, each evaluating 11 cases at two time points. The validity study was based on the content validation of 15 palliative care experts conducted over telephone interviews, with discussion on five themes: PPS as clinical assessment tool, the usefulness of PPS, PPS scores affecting decision making, the problems in using PPS, and the adequacy of PPS instruction.

**Results:**

The intraclass correlation coefficients for absolute agreement were 0.959 and 0.964 for Group 1, at Time-1 and Time-2; 0.951 and 0.931 for Group 2, at Time-1 and Time-2 respectively. Results showed that the participants were consistent in their scoring over the two times, with a mean Cohen's kappa of 0.67 for Group 1 and 0.71 for Group 2. In the validity study, all experts agreed that PPS is a valuable clinical assessment tool in palliative care. Many of them have already incorporated PPS as part of their practice standard.

**Conclusion:**

The results of the reliability study demonstrated that PPS is a reliable tool. The validity study found that most experts did not feel a need to further modify PPS and, only two experts requested that some performance status measures be defined more clearly. Areas of PPS use include prognostication, disease monitoring, care planning, hospital resource allocation, clinical teaching and research. PPS is also a good communication tool between palliative care workers.

## Background

The Palliative Performance Scale (PPS) was first introduced by Anderson and Downing in 1996 as a new tool for measurement of performance status in palliative care [1]. Its initial uses in Victoria, British Columbia included communication, analysis of home nursing care workload, profiling admissions and discharges to the hospice unit and prognostication. PPS has been used in many countries and has been translated into other languages, including French, Japanese, German and Thai (from private correspondence of Downing GM, 2008). In a recent systematic review on clinical assessment tools for quality of life, Jordhoy et al [2] found that out of the 39 existing palliative care assessment tools, 11 included original performance status assessments, and only two of them, PPS and Edmonton Functional Assessment Tool (EFAT) incorporated comprehensive performance status measures. PPS is observer-rated and includes five domains (on a Scale of 0% to 100%, in increments of 10%) – Ambulation; Self-care; Activity Level/Evidence of Disease; Intake and Level of Consciousness (Additional file 1). It is adapted from the Karnofsky Performance Scale [3].

A brief review of the literature showed only a dozen articles on PPS, including the original 1996 Anderson article where its development was first reported. Only seven of these articles are on the validity of PPS in prognostication and its usefulness in the prediction of survival of palliative care patients. Five of these articles are in peer-reviewed journals (Morita [4] 1999; Virik [5] 2002; Harrold [6] 2005; Head [7] 2005; Lau [8] 2007), with the other two being conference presentations and abstracts (Younis [9] 2003; Olajudin [10] 2004). Other documented areas where PPS are used include disease progress monitoring and care planning. In the Canadian Health Services Research Foundation report by Dudgeon, on Collaborative Care Plans [11], PPS was one of the assessment tools recommended. In another article on advance planning and palliative care in nursing homes in USA, Levy also used PPS for prognostication and palliative care interventions [12]. The "BC Palliative Care Benefit Program (2001)" of British Columbia, Canada, contains a guideline for their Drug Benefit Program, stating that eligible patients must be in the terminal stage of illness with an entry point PPS of 50% level or below [1].

Downing et al [13] in their meta-analysis study of PPS, found only Harrold [6] reported interrater reliability test results for PPS in a pilot study with a small sample size (n = 30). As PPS is becoming more widely used in palliative care, and no formal study has been done on its inter- and intrarater reliability and validity, it is important for clinicians to have solid evidence that PPS % level scores can be repeated consistently and also accurately reflect the functional status of the patients assessed.

This study has two objectives: (1) to examine the inter- and intrarater reliability of the PPS by clinicians, (2) to examine the validity of the PPS by content validation, questioning clinicians on its purpose and usefulness.

## Methods

This study obtained ethics approval for human participant research, by the Human Research Ethics Board, University of Victoria, BC (Protocol Number 07-05-385b), and has met the standards of research design, and the protection of rights and security of data.

### Reliability study

The reliability study was designed as a web-based scenarios study using a secure website, initially with 22 simulated palliative care patient case histories created from actual clinical settings (see Additional file 2 for a sample case). Because the time required to evaluate all 22 cases was too long, we randomly divided the cases into two groups: 1 and 2, with 11 cases in each. When a participant first registered, a random number, either 1 or 2 was generated by computer program, and the participant was assigned to the corresponding group. When a participant logged onto the website, he/she was presented with the PPS and its instructions of use. The cases were presented in a random order, so that no two participants in the same group read the cases in the same sequence. The participant was asked to review the history of each case, assign a PPS % level and go on to the next case, until all 11 cases were completed. This was Time-1 of the reliability study. Two weeks later, each participant was invited back to repeat the test in the same way, with the same 11 cases and the same sequence of presentation. This was Time-2 of the reliability study.

#### Case Development

The cases were in narrative format. Palliative care clinicians from hospices, in-hospital consultation services and palliative out-patient clinics provided us with their patient case histories. Cases were developed using the material obtained, modifying them somewhat to ensure anonymity, with the intention of covering all scenarios of the different PPS % levels, from PPS 10% to PPS 100%. A panel of palliative care experts, three physicians and three nurses (Case Development Experts), reviewed all of the cases at a face to face meeting. First each case was scored by the experts individually, then the score results were compared and the differences in PPS % levels (if any) were discussed, and with consensus, the narration/wordings of each case were adjusted to be consistent with a given PPS % level. There were 22 cases in total.

#### Participant Recruitment

Participants of the reliability study were recruited through a two step process. An email with an attached invitation to participate, was sent to the administrators and senior clinicians of various palliative care institutions across Canada and the U.S.A. (Victoria, Vancouver, Calgary, Edmonton, Toronto, Hamilton, Kingston, Montreal, Newfoundland, Nova Scotia and North Carolina), requesting that they forward the invitation to their members and colleagues. This invitation provided detailed information about the mechanics of the web-based study, the researchers involved, the anonymyzed format of the data obtained, participants' confidentiality, the time required for participants to complete the study and the steps in giving consent, and/or withdrawing from the study. Upon receiving the forwarded invitation, clinicians (palliative care physicians and nurses) responded by returning an 'acceptance to participate' email to the researchers, agreeing to participate in the study. A username and password were created for each participant and together with the URL of the secure website, were sent to them by email.

### Validity study

The validity study was performed through content validation interviews with palliative care experts, discussing their experiences using PPS. An email 'invitation to participate' was sent to clinicians (both physicians and nurses), who were experts in using PPS in their palliative care practice. Potential participants' names were obtained from suggestions by one of the authors of this study. Included in the invitation email was a list of five questions that would be discussed in a telephone interview. The questions were based on five themes: PPS as a clinical assessment tool, areas of usefulness of PPS, how the PPS scores affected clinicians' decision making in patient management, the problems in using PPS, and the adequacy of PPS instruction. Experts were also asked about the number of years they have been using PPS. The questions were approved by the authors of the study before they were sent out to the potential participants. For those who returned an acceptance email, arrangements were made for appropriate interview schedules. The experts recruited were from across Canada and the U.S.A. (Victoria, Vancouver, Edmonton, Toronto, Kingston, and Newfoundland and North Carolina). Each interview took approximately 20–30 minutes, notes were taken and the interviews were audio-recorded for cross-referencing. One author collected all the interview results and all opinion and suggestions were sorted under the five themes. These were presented, discussed and content-analyzed by all authors in a meeting, questions were raised and referred back to the recordings for clarification.

### Analytical Methods

The data obtained from the reliability portion of the study was analyzed using SPSS version 15.0 and R version 2.5 software. The reliability of PPS was evaluated using the single rating intraclass correlation coefficient (ICC) for absolute agreement and for consistency using two-way random-effects models. Single rating measures were used because our interest was in ICC measures for individual participants rather than averages of independent measures. The ICCs were obtained for each time period.

Participant reliability was evaluated using Cohen's kappa, which is a chance-corrected measure of agreement between two participants. It ranges from 0 (chance agreement) to 1 (perfect agreement), and generally a kappa > 0.7 is considered satisfactory. An interpretation by Landis and Koch [14] divided kappa into six categories: < 0 (no agreement), 0.0–0.20 (very low agreement), 0.21–0.40 (low agreement), 0.41–0.60 (moderate agreement), 0.61–0.80 (full agreement) and 0.81–1.00 (almost perfect agreement).

We looked at the results of individual cases, using boxplots. The top and bottom of the box represent the 75^th ^and 25^th ^percentiles respectively, a horizontal line across the box identifies the median, and the hinges on the top or bottom are the highest and lowest values excluding outliers. Outliers are depicted with circles, and are defined as values that extend from 1.5 to 3 box lengths below the 25^th ^percentile or above the 75^th ^percentile. We also compared participants' scores to the intended PPS % levels agreed upon by the Case Development Experts.

Content validity is based on the extent to which a measurement adequately and comprehensively reflects the specific intended domain of content, and it employs a reference standard. In our study, we questioned the experts regarding the usefulness and problems of PPS. The reference standard we used was the actual experience of the experts in the domain of palliative care. During our telephone interviews with palliative care experts, notes were taken and the discussions were audio-recorded. Content analysis of the interviews was based on the notes taken, cross referenced with the audio-recording when required and the results were grouped into five themes for reporting.

## Results

### Reliability study

#### Descriptive Analysis

A total of 62 emails were sent to administrators and senior clinicians of palliative care institutions, requesting them to forward our "Invitation to Participate" letter to their colleagues and members. We do not have a record of which administrators and senior clinicians actually forwarded our letter, but we did have a total of 73 individuals who returned a 'consent to participate' email. Out of these participants, 65 completed Time-1, and among them, 53 also completed Time-2. Only scores of participants who completed both Time-1 and Time-2 were included in this study.

The participants were randomly assigned to Group 1 or Group 2, each with 11 cases. There were 25 participants in Group 1 and 28 in Group 2. By design, the range of PPS % levels of all the 22 cases (both Group 1 and Group 2) covered the entire spectrum of values, from PPS 10% to PPS 100%. Because the 22 cases were randomly allocated to either Group 1 or Group 2 (each with 11 cases) when the study was first set up, Group 1 ended up with no representation at PPS 20% and PPS 80% and Group 2 with no PPS 40%, 70% and 100%.

**Figure 1:**
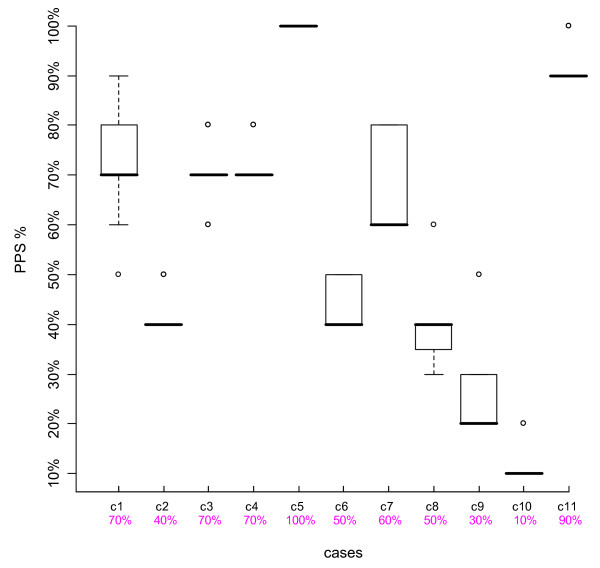
**Boxplot Group 1 Time-1**. The Y-axis represents the participants' PPS % levels, the top line X-axis shows the individual case number, and the 2^nd ^line X-axis gives the intended PPS % levels as determined by the Case Development Experts.

**Figure 2:**
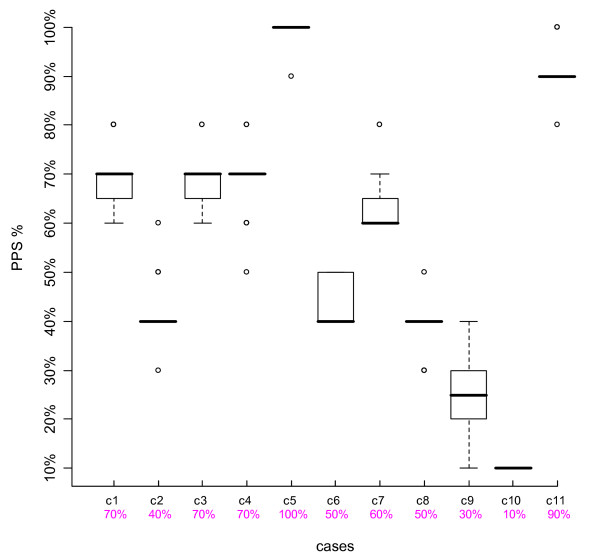
**Boxplot Group 1 Time-2**. The Y-axis represents the participants' PPS % levels, the top line X-axis shows the individual case number, and the 2^nd ^line X-axis gives the intended PPS % levels as determined by the Case Development Experts.

**Figure 3:**
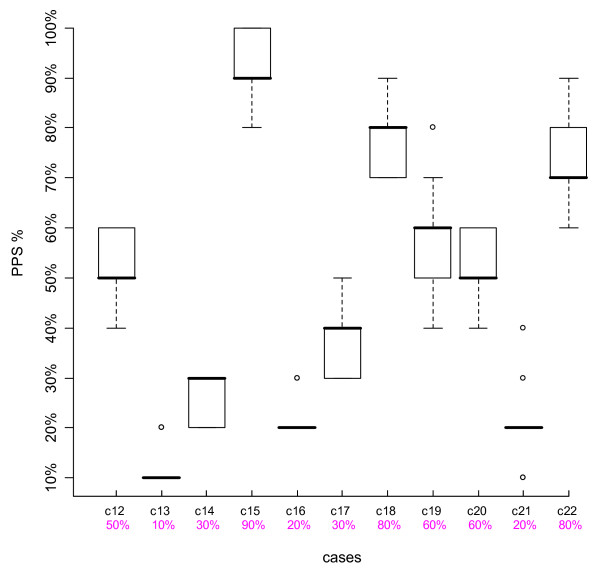
**Boxplot Group 2 Time-1**. The Y-axis represents the participants' PPS % levels, the top line X-axis shows the individual case number, and the 2^nd ^line X-axis gives the intended PPS % levels as determined by the Case Development Experts.

**Figure 4:**
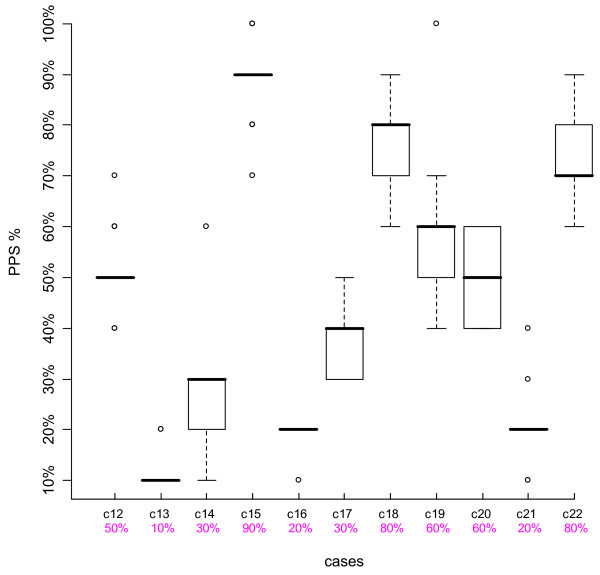
**Boxplot Group 2 Time-2**. The Y-axis represents the participants' PPS % levels, the top line X-axis shows the individual case number, and the 2^nd ^line X-axis gives the intended PPS % levels as determined by the Case Development Experts.

Figures 1, 2, 3 and 4 are boxplots of the PPS % scores by case for the two time periods for both Group 1 and Group 2. The variation is quite small for most cases, within 0–2 PPS % levels, indicating consistency in scoring. Outliers were present in about half of the cases, not necessarily at the same PPS % levels or with the same case for both Time-1 and Time-2. The second row of numbers on the x-axis of the boxplots represents the intended PPS % level of each case as determined by the Case Development Experts. In each Figure, for eight out of the 11 cases, the intended PPS % levels were the same as the medians of the boxplots and for the remaining three cases, in all but one Figure, the intended PPS % levels fall within the 75^th ^and 25^th ^percentiles of each boxplot. Over all of the participant and case scores, 67.6% were the same as those intended by the Case Development Experts. Thus there is strong agreement between the Case Development Experts and the participants.

#### Reliability Analysis

The reliability of PPS was calculated using ICC for absolute agreement and for consistency using two-way random-effects models. ICC measures the proportion of variance in the measurements attributable to the cases. Larger values, near 1, indicate that most of the variation in the measurements is due to the differences in the cases, rather than to variability between participants. Values were obtained for each time period for the two groups. Absolute agreement measures take into account participant variability, while measures of consistency do not. Table 1 summarizes the reliability results. The consistency and absolute agreement values are very similar and high, around 0.96, indicating good agreement among participants.

**Table 1:** PPS – ICC

		Consistency	Absolute Agreement
Group	Time Period	Intraclass Correlation (95%CI)	Intraclass Correlation (95%CI)

1 (n = 28)	1	.960 (.919, .987)	.959 (.917, .986)
1	2	.963 (.926, .988)	.964 (.927, .988)
2 (n = 25)	1	.959 (.917, .986)	.951 (.901, .984)
2	2	.938 (.878, .979)	.931 (.864, .977)

Reliability Results – Intraclass Correlations and their 95% Confidence Intervals

##### Participants (raters) Reliability

Table 2 shows a cross tabulation of PPS % levels at Time-1 and Time-2, Group 1 and 2 combined. The x-axis represents PPS % levels of participants at Time-2, and the y-axis represents PPS % levels of participants done at Time-1. The least amount of deviation occurred at the two ends of the PPS % levels, i.e. PPS 10% and PPS 100%, suggesting that it is easier to score the same value (PPS 10% and PPS 100%) in both Time-1 and Time-2, when patients were either healthy or very sick. Overall 74.4% of the scores were on the diagonal, indicating most participants scored with the same values in both Time-1 and Time-2.

**Table 2:** Cross Tabulation of Time-1 & Time-2 Scores

Time-1						Time-2					
Level	10%	20%	30%	40%	50%	60%	70%	80%	90%	100%	Total

10%	**51**	0	0	0	0	0	0	0	0	0	51
20%	5	**57**	11	1	0	1	0	0	0	0	75
30%	0	10	**30**	4	0	0	0	0	0	0	44
40%	0	0	4	**65**	9	1	0	0	0	0	79
50%	0	0	0	16	**30**	3	0	0	0	0	49
60%	0	0	0	1	6	**41**	4	2	0	1	55
70%	0	0	0	0	1	18	**53**	8	0	0	80
80%	0	0	0	0	0	6	15	**26**	1	0	48
90%	0	0	0	0	0	0	1	5	**34**	3	43
100%	0	0	0	0	0	0	0	0	7	**32**	39

Total	56	67	45	87	46	70	73	41	42	36	563

Time 1 vs Time 2 PPS % levels of all 22 cases.

The X-axis represents the Time 2 scores and Y-axis represents the Time 1 scores.

The values in the table represent the total number of scores at each PPS % level at Time 1 and Time 2.

##### Cohen's kappa

Table 3 lists the summary statistics for Cohen's kappa, calculated over all of the participants, for Time 1 and Time 2. Group 1 has a mean kappa of 0.67 and Group 2 has a mean kappa of 0.71, indicating satisfactory results among participants.

**Table 3:** Cohen's Kappa Statistics

**mean**	**median**	**max**	**min**
*Group 1*			
0.6680388	0.6379710	1.0000000	0.2735849
*Group 2*			
0.7099952	0.6886792	1.0000000	0.3592233

### Validity study

In this study, we conducted content validation through expert opinions. The 15 experts interviewed included both physicians and nurses working in the field of palliative care as in-hospice clinicians, members of palliative care home teams, hospital palliative consultants, physicians of palliative out-patient clinics and physicians at cancer institutes. They are from across Canada and U.S.A. (British Columbia, Alberta, Ontario, Newfoundland and North Carolina). All of them have used PPS for more than two years; and one expert has used it since it was first introduced over ten years ago.

The interviews were conducted, content analyzed and grouped into five themes. The experts' opinions were summarized as follows.

#### i. PPS as a clinical assessment tool

The experts agreed that PPS is a valuable tool in the clinical assessment of all palliative care patients, not just end-of-life patients. Some wanted to apply the usage of PPS to other domains such as pediatric patients, acute in-hospital patients or even suggested teaching patients and their families how to use PPS, to assist them in making end of life decisions. PPS is one of the standard assessment tools in many experts' palliative care practices; although one expert uses ECOG (Eastern Cooperative Oncology Group Performance Status) with his oncology patients and uses PPS only with his other patients, other experts use PPS with all their palliative care patients. One other expert pointed out that PPS is helpful in tracking a disease trajectory, especially when a patient's condition is deteriorating.

#### ii. Areas of usefulness of PPS

Areas of use are emphasized differently, depending on the type of practice of the experts. Patients of hospital palliative consultants are in quicker transition, and by the time they are consulted, patients are usually at the end-stage of life. In these situations with PPS levels of 10%–20%, prognostication is a more important issue than disease monitoring. For in-hospice clinicians, patient disease monitoring during their end-of-life course in hospital, is as important as prognostication. For hospital palliative care administrators, patient care planning and hospital resource allocation (e.g. hospital beds), become priorities. A detailed description of patients in the various areas of their palliative care program, using PPS as the indicator of patients' state, can help in administrative planning. Palliative care home nurses frequently use PPS to evaluate which patients require home nursing care. PPS is a useful tool for teaching residents to include patient function in their assessment of whether a patient should be discharged from hospital.

#### iii. How PPS scores affect clinicians' decision making in patient management

As one expert pointed out, PPS is valuable in placement, but not in treatment decision making. Clinicians are trained to base treatment decisions on patient symptoms. On the other hand, PPS can assist family members in making tough end-of-life decisions. PPS is also helpful in decision in placement of patients, from active care to palliative care.

#### iv. Problems using PPS

Problems raised by the experts can be categorized as: (1) on PPS performance status (2) PPS % level scores (3) PPS learning curve and (4) PPS as communication tool. Many experts felt that PPS should be left as is; however, two experts thought some performance status indicators needed to be further defined. For example, ambulation should specify the length of time that a patient can sit in a chair. The term "oral intake" should be redefined to include other types of intake, for example, NG/PEG tube feeding and supplements. The scoring of comatose patients is unclear, and the meaning of "extensive disease" is more difficult to define in cases of COPD or stroke.

Some experts found it more difficult to score at certain PPS % levels. One expert asked what to do with PPS % level scores that fell in between two levels and requested finer grading of PPS % levels. One expert found it easier to score with lower PPS % levels, and more difficult with higher levels. Other experts found it troublesome to distinguish between certain PPS % levels, e.g. between PPS 30% and 40%, or between PPS 80% and 90%. In a tertiary care setting, patients' PPS % level are usually above PPS 60%, and those experts had less experience dealing with lower PPS levels.

One expert commented that if PPS % levels were used in disease trajectory monitoring, the trajectory shape, linear or nonlinear could depend on how frequently PPS % level was recorded. For example, a patient admitted with PPS 10%, may be at PPS 40% after treatment, then drop back to 10% within a few days. If PPS % levels were only taken at both ends of this time period, a linear graph would result, with no change in PPS % level. But if the PPS % levels were taken more frequently, then a curve would be obtained.

One expert who used PPS only for his non-cancer patients found that PPS had too many variables to remember, so that he frequently referred back to the PPS table and required PPS instructions while using the tool, while others who used PPS regularly had no such problem.

Experts agreed the learning curve for PPS is somewhat steep, but once it is used properly, PPS is a good communication tool and becomes a standard reference for palliative care workers to discuss patients' conditions among themselves. One expert suggested, "Sometimes it is laziness that prevents palliative care workers from understanding how to use PPS."

#### v. Adequacy of PPS instruction

Some found the guideline was very useful but too long to review and it was sometimes difficult to find detailed information in the descriptions. One expert suggested moving some of the instructions to the actual PPS table. Another expert requested more case examples be included.

## Discussion

### Reliability

PPS is an important clinical assessment tool in palliative care, and its indicators are based on the performance status of the patient. It has been used in prognostication, disease progress monitoring, in administration and health care planning. It is reliable with ICC values for consistency and absolute agreement around 0.96. From Table 2, the Cross Tabulation of Time-1 and Time-2 Scores, the PPS % levels are consistently scored the same over the two time periods. When comparing the participants' PPS % levels with the intended PPS % levels set by the Case Development Experts, the agreement was very good; demonstrating that PPS can produce consistent results among users and that PPS is a reliable tool.

Cohen's kappa was used to measure interrater reliability of participants over time, with a mean kappa of 0.67 in Group 1 and 0.71 in Group 2. Because there were only 11 cases evaluated by each participant, kappa values were greatly influenced by any scores that were not the same over the two time periods. With a larger number of cases, we would expect greater test-retest reliability.

The boxplots of PPS scores demonstrated that outliers were present. This phenomenon can be explained as a built-in problem of the study design. Participants based the PPS scores on the text narration of the case history. For some cases, a performance status could be determined from only a few words about a patient's condition. If those words or description were overlooked, a different level would be assigned. In an actual clinical setting, each performance evaluation is based on the observation of the patient, and if the clinician is unsure of the patient's function, he/she can re-examine a patient repeatedly to arrive at a satisfactory PPS % level. This minimizes the chance of outliers. Also PPS is usually used as a communicative tool, where palliative care workers can come to consensus of a PPS % level, as the basis for discussion about an individual patient.

### Validity

The validity study was based on content validation using interviews with palliative care experts, on different aspects of PPS usage. All of the experts agreed that PPS is a valuable clinical assessment tool in palliative care and many of them have already incorporated PPS as standard in their practice. Most did not feel a need to further modify PPS, with the exception of two experts who suggested fine tuning to better define some performance status indicators, specifically oral intake and mobility.

Difficulty can be found in two areas: scoring a PPS % level and in learning how to use PPS. Some experts found occasional uncertainty in differentiating between PPS % levels of 30% and 40%, and also between PPS % levels 80% and 90%. Others were not sure about the 'in-between' values of two adjacent PPS % levels, although it is strongly emphasized in the PPS instruction manual, to utilize the 'best fit' method in determining the best horizontal PPS % level. Some PPS users may not read the instructions carefully enough to extract this intent.

Does this mean the PPS scale should be sub-divided into finer grading? There will always be 'in-between' values; no matter how finely tuned the grading system is. The balance between ease of use with fewer scores and complexity of using more line items was weighed in favour of the former. Trying to remember 22 lines versus 11 would reduce the utility, and thus the 'best fit' remains the approved standard. Although the learning curve for PPS is initially difficult for some, its ease of use (including differentiating between certain PPS % levels) comes with practice and experience.

The use of PPS in prognostication has been studied by a few researchers [6-8] over the past ten years. PPS is also used in decision making in hospital administration planning and resource allocation and PPS is used as a qualifier for admission into a drug benefit program [15] and home nursing care programs. It is also useful in disease monitoring, clinical teaching and research. Such multiple purposes strengthen its validation.

Among hospital and hospice palliative care workers, PPS is a good communication tool for discussing patients' condition. For a given PPS % level, everyone will know what condition the patient is in, and has a clear concept of the performance statuses that come with that particular PPS % level. PPS simplifies and enhances communication.

In summary, the palliative care experts agreed that PPS is valuable in clinical assessment, and has been integrated into their standard of practice. Although a few raised the question of modifying the performance status measures, most felt PPS should be left as is. Although it requires time to learn initially, but once mastered, it is very valuable in communication.

## Conclusion

This article has described the reliability testing and validity study of PPS, a clinical performance assessment tool used in palliative care patient management. The reliability testing showed PPS is a reliable tool. Our validity study was based on the content validation of palliative care experts, and they all agreed that PPS is valuable in the clinical assessment of palliative care patients.

## Competing interests

The authors declare that they have no competing interests.

## Authors' contributions

FH developed the website for the reliability study, composed the 22 web-based cases with consultation with the Case Development Experts and conducted the interviews in the validity study. FL, MGD and ML contributed to the design of the study and the analysis of the data; MGD was also consulted on the development of the cases; ML performed the statistical analyses. All authors read and approved the final manuscript.

## Pre-publication history

The pre-publication history for this paper can be accessed here:



## Supplementary Material

Additional File 1 Appendix A. Palliative Performance Scale. Click here for file

Additional File 2 Appendix B. PPS Reliability Study Sample Cases. Click here for file
