# Supplementary material for: A reliability and validity study of the Palliative Performance Scale
Source: BMC Palliat Care. 2008 Aug 4;7:10. doi: 10.1186/1472-684X-7-10 (PMC2527603; doi:10.1186/1472-684X-7-10)
Supplement: Additional File 1 — Appendix A. Palliative Performance Scale. [file 1472-684X-7-10-S1.docx]

# Additional files

## **Appendix A – Palliative Performance Scale**

| PPS Level | Ambulation | Activity & Evidence of Disease | Self-Care | Intake | Conscious Level |
| --- | --- | --- | --- | --- | --- |
| 100% | Full | Normal activity & work No evidence of disease | Full | Normal | Full |
| 90% | Full | Normal activity & work Some evidence of disease | Full | Normal | Full |
| 80% | Full | Normal activity with Effort Some evidence of disease | Full | Normal or reduced | Full |
| 70% | Reduced | Unable Normal Job/Work Significant disease | Full | Normal or reduced | Full |
| 60% | Reduced | Unable hobby/house work Significant disease | Occasional assistance necessary | Normal or reduced | Full  or Confusion |
| 50% | Mainly Sit/Lie | Unable to do any work Extensive disease | Considerable assistance required | Normal or reduced | Full  or Confusion |
| 40% | Mainly in Bed | Unable to do most activity Extensive disease | Mainly assistance | Normal or reduced | Full or Drowsy  +/- Confusion |
| 30% | Totally Bed Bound | Unable to do any activity Extensive disease | Total Care | Normal or reduced | Full or Drowsy  +/- Confusion |
| 20% | Totally Bed Bound | Unable to do any activity Extensive disease | Total Care | Minimal to  sips | Full or Drowsy  +/- Confusion |
| 10% | Totally Bed Bound | Unable to do any activity Extensive disease | Total Care | Mouth care  only | Drowsy or Coma  +/- Confusion |
| 0% | Death | - | - | - | - |
