# Supplementary material for: A reliability and validity study of the Palliative Performance Scale
Source: BMC Palliat Care. 2008 Aug 4;7:10. doi: 10.1186/1472-684X-7-10 (PMC2527603; doi:10.1186/1472-684X-7-10)
Supplement: Additional File 2 — Appendix B. PPS Reliability Study Sample Cases. [file 1472-684X-7-10-S2.docx]

# Additional files

**Appendix B – PPS Reliability Study Sample Cases**

CASE #1

Mrs. H. is a 68 year old woman whose main support persons are her husband and daughter. She had adenocarcinoma of the sigmoid colon 10 years ago, surgically resected, and was disease free until last year, with metastases to her lungs. The oncologist is recommending chemotherapy which she is thinking about but quite hesitant. She resides at home and with the help of home support worker and home care nurse. She does her own personal care after a caregiver helps her to the bathroom. She moves slowly but is steady on her feet. At night, she uses a commode at the bedside. She spends much of the day sitting in a lazy-boy chair in the living room and falls asleep. She frequently nods off but awakens readily when roused

She has only one good meal a day and finishes at best, about 30% of that. She experiences pain in her right chest and lower abdomen, helped with regular analgesics. She complains of hesitancy with urination and occasional diarrhea and has periods of profuse sweating. Her left leg is edematous and her abdomen distended

(PPS Score 50%)
